# Mining and visualizing high-order directional drug interaction effects using the FAERS database

**DOI:** 10.1186/s12911-020-1053-z

**Published:** 2020-03-18

**Authors:** Xiaohui Yao, Tiffany Tsang, Qing Sun, Sara Quinney, Pengyue Zhang, Xia Ning, Lang Li, Li Shen

**Affiliations:** 1 Department of Biostatistics, Epidemiology and Informatics, Perelman School of Medicine, University of Pennsylvania, Philadelphia, PA, 19104 USA; 2 Department of Computer and Information Science, School of Engineering and Applied Science, University of Pennsylvania, Philadelphia, PA, 19104 USA; 3 Department of Obstetrics and Gynecology, School of Medicine, Indiana University, Indianapolis, IN, 46202 USA; 4 Department of Biomedical Informatics, College of Medicine, Ohio State University, Columbus, OH, 43210 USA

**Keywords:** High-order drug interaction, Directional effect, FAERS, Apriori, Sunburst

## Abstract

**Background:**

Adverse drug events (ADEs) often occur as a result of drug-drug interactions (DDIs). The use of data mining for detecting effects of drug combinations on ADE has attracted growing attention and interest, however, most studies focused on analyzing pairwise DDIs. Recent efforts have been made to explore the directional relationships among high-dimensional drug combinations and have shown effectiveness on prediction of ADE risk. However, the existing approaches become inefficient from both computational and illustrative perspectives when considering more than three drugs.

**Methods:**

We proposed an efficient approach to estimate the directional effects of high-order DDIs through frequent itemset mining, and further developed a novel visualization method to organize and present the high-order directional DDI effects involving more than three drugs in an interactive, concise and comprehensive manner. We demonstrated its performance by mining the directional DDIs associated with myopathy using a publicly available FAERS dataset.

**Results:**

Directional effects of DDIs involving up to seven drugs were reported. Our analysis confirmed previously reported myopathy associated DDIs including interactions between fusidic acid with simvastatin and atorvastatin. Furthermore, we uncovered a number of novel DDIs leading to increased risk for myopathy, such as the co-administration of zoledronate with different types of drugs including antibiotics (ciprofloxacin, levofloxacin) and analgesics (acetaminophen, fentanyl, gabapentin, oxycodone). Finally, we visualized directional DDI findings via the proposed tool, which allows one to interactively select any drug combination as the baseline and zoom in/out to obtain both detailed and overall picture of interested drugs.

**Conclusions:**

We developed a more efficient data mining strategy to identify high-order directional DDIs, and designed a scalable tool to visualize high-order DDI findings. The proposed method and tool have the potential to contribute to the drug interaction research and ultimately impact patient health care.

**Availability and implementation:**

http://lishenlab.com/d3i/explorer.html

## Background

Recent advances in large-scale electronic health record database techniques provide exciting new opportunities to the study of drug safety. Drug-drug interactions (DDIs), a major cause of adverse drug events (ADEs), are a serious global health concern, and a severe detriment to public health. In fact, over 500,000 serious medical complications per year, a portion of which are fatal, result from multiple drug consumption [1]. The most common cause of ADEs is DDIs, and more than three-fourths of American elderly citizens take two or more drugs per day [2]. Therefore, studying DDIs is clearly a relevant and pressing area of research.

The scale of DDIs involving three or more drugs (also called high-order DDIs) has posed a prohibitory challenge for molecular pharmacology and clinical research, which motivates alternative strategies such as mining health record data. This project aims to develop large-scale computational strategies and effective software tools for mining high-order DDI effects from health record databases, in order to yield novel discoveries in drug safety, and ultimately to benefit national health and well being.

Although many research groups have used various statistical methods or machine learning algorithms to discover DDIs, most of these efforts have focused on finding pairwise DDIs [3]. Due to the lack of multiple-drug experimental data, a common method to predict high-order DDI is to piece together multiple pairwise analyses to form an overall high-order analysis [4]. However, studying high order DDI through pairwise analysis is a relatively simplistic approach, as it neglects the fact that networked interactions can change when a third drug enters the pair. For instance, a three-way combination could lead to ADEs even when its subsets of pairwise drug combinations do not [5]. DDIs get increasingly complex as more drugs are involved, and it is also statistically complicated to aggregate the results of separate pairwise analyses [2].

While investigation of high-order DDIs is still an under-explored area [2], new methods involving data mining have appeared to predict high-order DDIs, bypassing the need for experimental data. Ning et al. demonstrates that frequent drug combinations and clinical data indicating patient side effects can be extracted from public health record databases to find correlations between drug combinations and ADEs [3]. Ultimately, this becomes a binary classification problem of whether certain drug combinations lead to ADEs, like myopathy, a degenerative muscular condition.

However, high-order DDIs, or DDIs involving three or more drugs, is a topic that has only recently been researched. For instance, a recent article by Li [6] discusses how big data can drive the pharmacology research space, thus implying that this is a concept that has not yet been fully taken advantage of. Therefore, a logical next step in this research area is to improve on methods for discovering high-order DDIs. As the quantity of phenotypic and genomic data increases, we can use this big data opportunity to fine-tune statistical and machine learning algorithms to better predict high-order DDI effects. The research area of computationally finding DDIs is relatively new, and thus the development of novel approaches and analyses is a promising research direction. As ADE reporting data grows at an increasing rate, we are also facing challenges to properly analyze large datasets.

With the above observations, the goal of this study is to identify high-order DDIs via mining the FAERS, a public health record databases with 4,077,447 drug combination records of 1,763 drugs. The ADE of interest is myopathy, which is a muscular degenerative disorder. Our previous study [7] has reported directional effect of DDIs for myopathy, the results of which were limited to involve up to three drugs due to both computational time and space complexities. In this paper, a more efficient data mining strategy is utilized to extract all the high-order directional DDIs. Given increasing numbers of co-administrated drugs, the tree-structured visualization could not effectively show all the DDI findings for a high-order drug combination. Thus, the second novelty of this study is that we develop an efficient and scalable method to visualize high-order directional DDIs in an interactive and comprehensive manner.

## Methods

### Materials and data sources

The proposed strategy for detecting high-order directional DDI effects on ADEs was applied to a publicly available database, the FDA Adverse Event Reporting System (FAERS: https://open.fda.gov/data/faers/). Specifically, we apply our method on the myopathy event using ADE reporting records from FAERS, to investigate the directional effects of high-order DDI on myopathy.

Myopathy is a relatively frequent (around 3.64*%* in our dataset) and clinically important ADE, and has been listed as a side effect of more than 80 FDA approved drugs. Given its high frequency and close and complex associations of myopathy with drugs, it is appropriate to use myopathy-related events as testbed for investigating the performance of directional effects of high-order DDIs. Below, we describe the data preprocessing and present the summary statistics of the FAERS dataset we used in this study.

#### FAERS database

The data used for this analysis included reports from FAERS collected between Q1 2004 and Q3 2012. The FAERS is a database that contains information on adverse event and medication error reports submitted to FDA. Reports were obtained from the FAERS database, and preprocessed as described in [7]. Briefly, the most recent reports from each individual were extracted and organized as a list of records, where each record consisted of an ADE and corresponding administered drugs.

#### Myopathy-related case-control dataset

**Fig. 1:**
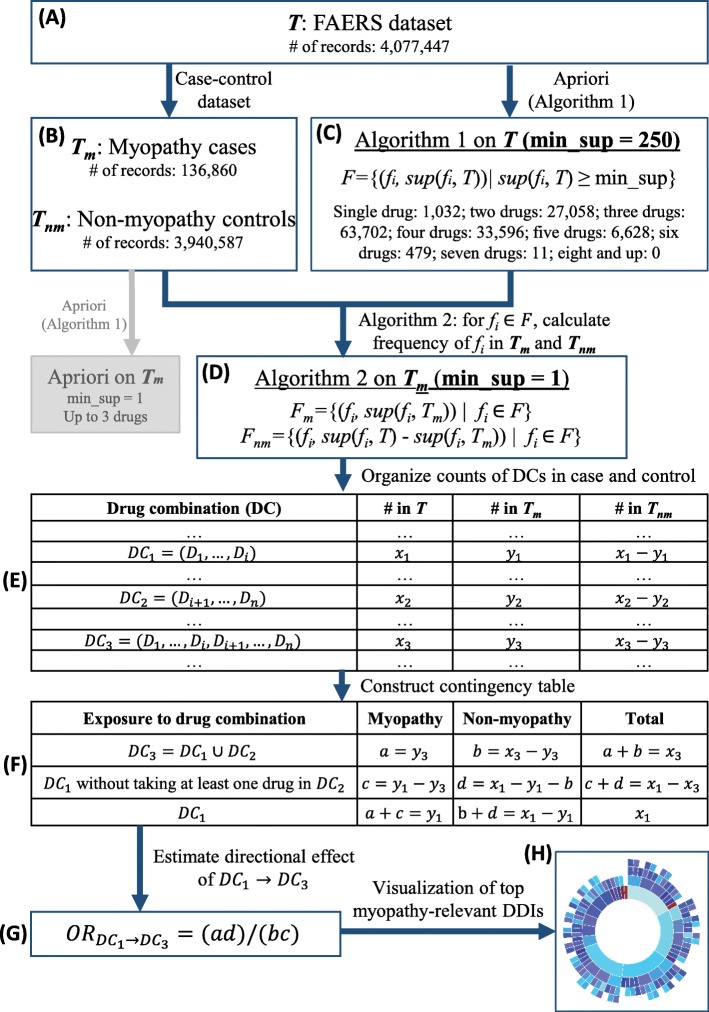
The workflow for mining high-order directional DDIs

As this analysis focused on the myopathy-related ADE, we firstly derived ADEs grouped under “myopathy”. And then we assembled a case-control dataset by labeling record as “case” if the ADE was in “myopathy” group, and otherwise labeling record as “control”. To avoid the confusion between causal effect and bystander effect, we included only drugs with primary or secondary suspects, while removing the drugs that were concomitant or interacting. We use *T* to denote the set of all the records from the FAERS database, and use *T*_*m*_ and *T*_*nm*_ to denote the sets of case and control records, respectively. Finally, totally |*T*|=4,077,447 records were analyzed, including |*T*_*m*_|=136,860 cases and |*T*_*nm*_|=3,940,587 controls, and totally 1,763 unique FDA approved drugs (see Fig. 1a-b).

The number of drugs contained in a single record ranges from 1 to 103, with a mean of 2.98 drugs in each record in FAERS. However, the numbers of drugs taken between two groups are significantly different (independent *T*-test *p*-value <2.2E-16), with mean of 4.18 drugs taken in myopathy cases and 2.94 drugs taken in non-myopathy controls. When focusing on records having more than three drugs, 25.82*%* individuals from FAERS dataset are taking four or more drugs together, while the proportion changes to 36.27*%* in myopathy cases and 25.45*%* in non-myopathy controls. Significant difference is also observed between these two groups (independent *T*-test *p*-value <2.2E-16), giving a mean of 8.79 drugs in myopathy group and 7.30 drugs taken in non-myopathy group.

### Methods for mining high-order directional dDI effects

We use *DC* to denote drug combination, and use *sup*(*DC*,*T*) to represent the support (i.e., count of occurrences) of *DC* in dataset *T*. To evaluate the risk of developing myopathy by adding drugs to existing drug combination, for example, taking *DC*_2_ = (*D*_*i*+1_,...,*D*_*n*_) in addition to taking *DC*_1_ = (*D*_1_,...,*D*_*i*_), we formulate the problem as follows: 1) the baseline population is defined as those who take *DC*_1_ = (*D*_1_,...,*D*_*i*_), regardless of taking other drugs or not; 2) exposed population is defined as those who take *DC*_2_ in addition to *DC*_1_, say *DC*_3_, where *DC*_3_ = *DC*_1_∪*DC*_2_; and 3) unexposed population is defined as those who take *DC*_1_ but without taking at least one drug from *DC*_2_. See Fig. 1e-f for a schematic example.

Then we employ the odds ratio (OR) to measure the directional DDI effect of adding *DC*_2_ to existing *DC*_1_, by formulating the DDI effect problem to mining the association between myopathy event with exposure to drug combination. In practice, the OR compares the odds of exposure to *DC*_2_ among cases to the odds of exposure to *DC*_2_ in controls, within the baseline population who all take *DC*_1_. Accordingly, given an interested drug combination, we need to calculate the number of exposed and unexposed population in both cases and controls before the calculation of OR.

In the following sections, we organize and present the framework as follows. First, we describe the algorithm for constructing candidate drug combinations. Second, we present the algorithm for extracting supports of occurrence of drug combinations in case and control datasets. After that, we discuss the calculation of OR for estimating the directional effect of drug combinations. Finally we present the novel and scalable tool we developed for visualizing high-order DDIs. Figure 1 shows the workflow of this study.

#### Construct candidate drug combinations from *T*

We first created a set of drug combinations with their supports from our FAERS dataset *T*. To avoid the possible misleading results from low-frequent drug combinations, we restricted our analysis to the *DCs* with a minimum support of *MinSup*=250 records in *T*, named candidate drug combinations. Algorithm 1 summarized the procedures for constructing candidate drug combinations from *T* (see Fig. 1c).

Briefly, we applied Apriori, an influential algorithm for mining frequent itemsets to *T*, to discover frequent *DCs* with *sup*(*DC*,*T*)>*MinSup* that involved up to seven drugs. Apriori has been used in our previous work [7] for mining the frequent drug combinations from both *T* and *T*_*m*_, using *MinSup*=1000 and *MinSup*=1 respectively. However, due to time and space complexities, our previous strategy could not generate drug combinations containing more than three drugs. Instead of applying Apriori on both *T* and *T*_*m*_, we only employed it on *T* to generate candidate *DCs*.


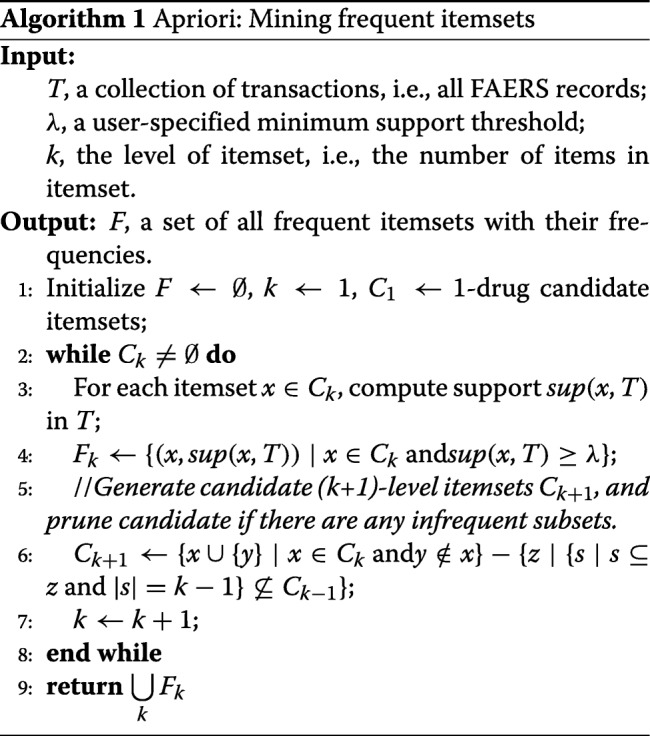


#### Computing supports for case records and control records

For each candidate drug combination obtained from above, we would like to extract their counts of occurrence in both cases and controls, for constructing contingency table for OR estimation. As we mentioned before, the computational time and space of using Apriori on *T*_*m*_ to extract DCs with *MinSup*=1 limited our previous work to involve up to three drugs (see gray part in Fig. 1). In this work, we develop a more efficient strategy to calculate supports for only candidate *DCs* instead of mining all possible drug combinations appeared in *T*_*m*_. Algorithm 2 describes how to extract the case and control supports from *T*_*m*_ and *T*_*nm*_ respectively (see Fig. 1d).


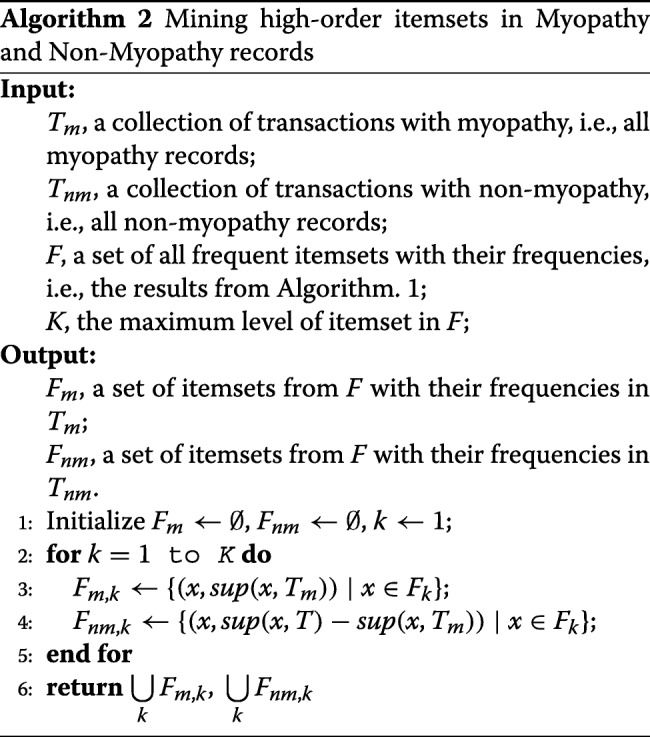


#### Estimating directional dDI effects

We organize the results from Algorithms 1 and 2, and construct a table of drug combinations (*DC*); see Fig. 1e. Each record in the table stores the counts of the corresponding *DC* in the entire studied FAERS set, the case subset and the control subset respectively. Base on this information, for each candidate *DC*, a contingency table is constructed and then used for OR calculation, where four counts *a*, *b*, *c* and *d* can be calculated as shown in Fig. 1f.

Figure 1e-g shows our procedure for estimating the directional effect of adding *DC*_2_ to *DC*_1_ on myopathy, including contingency table construction and OR calculation. The baseline population, exposed population and unexposed population are the sets of individuals who take *DC*_1_,*DC*_3_, and *DC*_1_ but without taking at least one drug in *DC*_2_, respectively. The numbers of exposed individuals with myopathy and non-myopathy can be directly extracted from Fig. 1e as follows: *a*=*y*_3_ exposed individuals with myopathy and *b*=*x*_3_−*y*_3_ exposed individuals with non-myopathy. The numbers of unexposed individuals with and without myopathy (i.e., *c* and *d*) can then be obtained by computing the difference between baseline and exposed populations. That is, unexposed individuals with myopathy are the individuals from baseline population but not in exposed population. Based on Fig. 1e, given *y*_1_ individuals in the baseline population with myopathy, the number of unexposed individuals with myopathy is *c*=*y*_1_−*a*=*y*_1_−*y*_3_. Similarly, the number of unexposed individuals with non-myopathy is *d*=(*x*_1_−*y*_1_)−*b*=(*x*_1_−*y*_1_)−(*x*_3_−*y*_3_).

With the above calculation, the OR estimation of directional effect of *DC*_1_ to *DC*_3_ on myopathy can be computed as follows:

1 $$ OR_{DC_{1}\rightarrow DC_{3}}=\frac{a/b}{c/d}=\frac{ac}{bd}.   $$

Here ORs of the ADE for adding one to seven drugs are examined in this study.

Chi-square test is used in this work to evaluate the significance of associations between drug combination and myopathy ADE, were p-value and confidence interval corresponding to each odds ratio are obtained. Multiple comparison correction is further performed using the Bonferroni strategy.

### Sunburst visualization for directional dDI findings

Another important aspect of DDI mining is the visualization. In our previous work, we proposed a tree structure to visualize the directional DDIs involving up to three drugs. However, the growth of the tree was exponential, making it infeasible to read for combinations involving four or more drugs. In this paper, we develop a novel tool to organize and visualize high-order directional DDIs using D3 sunburst diagram (https://d3js.org/).

Specifically, given a candidate drug combination **S** and a set **C** of all subsets of **S**, we organize the pair-wised relationship of elements **C** and arrange them into a series of circles in a hierarchical manner as shown in Fig. 1h. Each ring sector represents a drug combination, outer ring sectors radiated from which indicate the directional DDIs from inner to outer. The sector color indicates the effect size (i.e., OR value). In addition, we include the zooming function to enable more effective visualization via interactive exploration, where one can select a drug combination as baseline to (1) zoom in and see the details or (2) zoom out and see an overall picture.

## Results

### Data summary

There are totally *T*=4,077,447 records included in our processed FAERS dataset, involving 1,736 unique FDA-approved drugs, of which *T*_*m*_=136,860 records are myopathy cases and *T*_*nm*_=3,940,587 records are non-myopathy controls. Using *MinSup*=250 as the frequency threshold on *T*, we obtained 1,032 frequent single drugs, 27,058 frequent two drug combinations, 63,702 frequent three drug combinations, 33,596 frequent four drug combinations, 6,628 frequent five drug combinations, 479 frequent six drug combinations, and 11 frequent seven drug combinations after running Algorithm 1 on *T* (Fig. 1c). There are no frequent drug combinations that include more than seven drugs available in our dataset.

### Myopathy-associated high-order directional dDIs

We have reported directional DDI results for myopathy that contained up to three drugs with minimum support of 1000 in [7]. Given the fact that 25.81*%* individuals have taken more than three drugs together and the proportion increases to 36.27*%* in myopathy cases, in this paper, we extended our previous work to mining DDIs with higher-order and reported all the myopathy associated directional DDI findings based on a less stringent mininum support of *MinSup*=250. As a result, we discovered higher-order directional DDIs involving up to seven drugs. We describe our results in the following subsections.

#### Effects of high-order drug combinations vs baseline

Tables S1-S7 show the top 10 findings from one to seven drugs versus the baseline. The top drug from one drug versus baseline (Table S1) is “fusidic acid”, a bacteriostatic agent primarily on inhibiting Gram-positive bacteria, with OR=27.24. This means the odds of myopathy in individuals taking fusidic acid is 27.24 times higher than in individuals not taking it.

Fusidic acid has been reported frequently associated with myopathy upon co-administration with statins [8]. There is no frequent co-administration of fusidic acid with other drugs from our results under the threshold of *MinSup*=250. However, we observe that the top two most frequent drugs taken together with fusidic acid in myopathy cases are both statins (simvastatin and atorvastatin). Specifically, there are 352 myopathy cases that take fusidic acid, among which 188 cases have taken both fusidic acid and simvastatin and 141 cases have taken both fusidic acid and atorvastatin. The counts of taking fusidic acid with these two stains decrease to 51 and 15 in non-myopathy controls, ranking as the 4*th* and 42*nd* co-administrated ones with fusidic acid. These indicate the joint effect of fusidic acid with statins on myopathy.

The use of *MinSup*=250 allows us to identify more myopathy risk DDIs than our prior study [7], consequently to help provide more comprehensive references for adverse effects of DDIs. For example, in addition to fusidic acid, another six drugs which have not been reported in our previous study are identified in this analysis, including telbivudine, cerivastatin, trabectedin, terconazole, flucloxacillin and pindolol (see Table S1). Tables S1-S7 summarize all the top 10 findings.

#### Directional effects of four-drug combinations

As mentioned above, our findings include the DDIs involving up to seven drugs. For the sake of conciseness, as an example, here we focus on reporting the top DDI results related to four-drug combinations. We calculated the ORs of the myopathy risk associated with the directional DDIs of each four drug combination versus all of its subsets. We obtained 27,112; 85,557; 87,069; and 25,575 significant findings with OR>1 for 4-drug combination versus baseline; 1-drug combination; 2-drug combination; and 3-drug combination, respectively. Shown in Tables 1, 2, 3 and 4 are the top 10 findings for the above four categories respectively.

**Table 1:** Top 10 OR results for 4-drug combination vs. baseline: with Bonferroni correction, a significant *p* is 1.50E-6

4-drug combination	OR	*p*-value
Fentanyl, Gabapentin, Levofloxacin, Zoledronate	49.65	6.46E-194
Furosemide, Gabapentin, Levofloxacin, Zoledronate	48.43	2.66E-174
Azithromycin, Ciprofloxacin, Levofloxacin, Zoledronate	45.30	2.27E-157
Azithromycin, Gabapentin, Levofloxacin, Zoledronate	44.97	4.17E-159
Gabapentin, Levofloxacin, Omeprazole, Zoledronate	44.76	4.47E-180
Gabapentin, Levofloxacin, Zoledronate, Zolpidem	44.74	2.77E-214
Fentanyl, Levofloxacin, Omeprazole, Zoledronate	44.62	6.06E-181
Gabapentin, Levofloxacin, Pamidronate, Zolpidem	44.37	1.10E-179
Alprazolam, Levofloxacin, Omeprazole, Oxycodone	43.24	1.45E-151
Gabapentin, Levofloxacin, Omeprazole, Oxycodone	42.44	8.54E-160

**Table 2:** Top 10 OR results for 4-drug combination vs. 1-drug: with Bonferroni correction, a significant *p* is 3.75E-7

4-drug combination	1-drug combination	OR	*p*-value
Fentanyl, Gabapentin, Levofloxacin, Zoledronate	Fentanyl	43.30	6.52E-181
Fentanyl, Levofloxacin, Omeprazole, Zoledronate	Fentanyl	38.79	2.03E-168
Ciprofloxacin, Fentanyl, Levofloxacin, Zoledronate	Fentanyl	36.70	1.15E-157
Acetaminophen, Doxorubicin, Omeprazole, Zoledronate	Doxorubicin	35.40	4.44E-114
Capecitabine, Dexamethasone, Fentanyl, Zoledronate	Capecitabine	34.36	9.61E-100
Alprazolam, Fentanyl, Levofloxacin, Zoledronate	Fentanyl	34.33	1.19E-146
Acetaminophen, Doxorubicin, Levofloxacin, Zoledronate	Doxorubicin	33.53	4.23E-113
Ciprofloxacin, Fentanyl, Levofloxacin, Oxycodone	Fentanyl	33.40	2.10E-146
Fentanyl, Gabapentin, Metoclopramide, Zoledronate	Metoclopramide	33.13	4.86E-117
Docetaxel, Oxycodone, Prochlorperazine, Zoledronate	Docetaxel	32.97	1.57E-140

**Table 3:** Top 10 OR results for 4-drug combination vs. 2-drug: with Bonferroni correction, a significant *p* is 2.61E-7

4-drug combination	2-drug combination	OR	*p*-value
Gadobenate Dimeglumine, Gadodiamide, Gadoteridol, Prednisone	Gadobenate Dimeglumine, Prednisone	270.16	8.81E-09
Pamidronate, Sulfamethoxazole, Trimethoprim, Zoledronate	Pamidronate, Sulfamethoxazole	85.71	4.87E-23
Doxorubicin, Pamidronate, Vincristine, Zoledronate	Doxorubicin, Vincristine	41.74	2.02E-129
Dexamethasone, Doxorubicin, Oxycodone, Vincristine	Doxorubicin, Vincristine	36.77	2.59E-92
Dexamethasone, Doxorubicin, Pamidronate, Vincristine	Doxorubicin, Vincristine	30.99	5.38E-108
Dexamethasone, Doxorubicin, Vincristine, Zoledronate	Doxorubicin, Vincristine	30.52	6.37E-112
Acetaminophen, Diphenhydramine, Prochlorperazine, Zoledronate	Diphenhydramine, Prochlorperazine	29.15	7.61E-62
Docetaxel, Oxycodone, Prochlorperazine, Zoledronate	Docetaxel, Prochlorperazine	28.95	9.23E-79
Acetaminophen, Cyclophosphamide, Doxorubicin, Pamidronate	Cyclophosphamide, Doxorubicin	27.76	1.79E-85
Docetaxel, Furosemide, Oxycodone, Zoledronate	Docetaxel, Furosemide	27.32	5.22E-58

**Table 4:** Top 10 OR results for 4-drug combination vs. 3-drug: with Bonferroni correction, a significant *p* is 5.27E-7

4-drug combination	3-drug combination	OR	*p*-value
Acyclovir, Dexamethasone, Pamidronate, Zoledronate	Acyclovir, Dexamethasone, Pamidronate	126.35	9.82E-29
Dexamethasone, Lorazepam, Thalidomide, Zoledronate	Dexamethasone, Lorazepam, Thalidomide	88.15	4.16E-22
Acetaminophen, Azithromycin, Cephalexin, Lorazepam	Azithromycin, Cephalexin, Lorazepam	77.91	6.75E-19
Docetaxel, Fentanyl, Oxycodone, Zoledronate	Docetaxel, Fentanyl, Oxycodone	75.67	7.06E-20
Pamidronate, Sulfamethoxazole, Trimethoprim, Zoledronate	Pamidronate, Sulfamethoxazole, Trimethoprim	70.29	2.35E-19
Docetaxel, Oxycodone, Prochlorperazine, Zoledronate	Docetaxel, Oxycodone, Prochlorperazine	62.64	2.99E-36
Acetaminophen, Dexamethasone, Oxycodone, Tramadol	Dexamethasone, Oxycodone, Tramadol	57.39	1.31E-14
Dexamethasone, Epoetin Alfa, Omeprazole, Zoledronate	Dexamethasone, Epoetin Alfa, Omeprazole	56.20	1.37E-32
Acetaminophen, Alendronate, Omeprazole, Oxycodone	Alendronate, Omeprazole, Oxycodone	53.65	2.97E-15
Acetaminophen, Fentanyl, Fluconazole, Oxycodone	Fentanyl, Fluconazole, Oxycodone	51.58	1.11E-14

Table 1 shows the top 10 findings of directional effects of adding four drugs to baseline, with OR values ranging from 42.44 to 49.65. There are twelve unique drugs across top 10 findings in Table 1, a number of which are indicated for pain relief. Specifically, fentanyl and oxycodone are opioid analgesics; gabapentin is a non-opioid analgesic and has been used in treatment of neuropathic pain; pamidronate and zoledronate are both bisphosphonates which are primarily used in treatment of bone metastasis. The top result illustrates that the risk of myopathy development would increase to 49.65 when taking fentanyl, gabapentin, levofloxacin and zoledronate together compared with baseline.

Based on Table 2, adding gabapentin, levofloxacin and zoledronate on top of fentanyl would result in 43.3 times altered risk of myopathy. Most of the top findings in Table 2 involve co-administration of zoledronate with different types of drugs including antibiotics (ciprofloxacin, levofloxacin), analgesics (acetaminophen, fentanyl, gabapentin, oxycodone) and others. Among the above mentioned drugs, several are reported to increase myopathy risk as single agents. However, to the best of our knowledge, no previous reports have indicated the interactions among these drugs. We will discuss these findings in the next section.

Tables 3 and 4 present the top OR results of comparing four-drug combinations with their subsets of two- and three-drug combinations. Part of these findings are similar to those in Tables 1 and 2. For example, adding zoledronate to the combination of docetaxel, oxycodone and prochlorperazine increases the risk of myopathy with OR=62.64 (see the 6th findings in Table 4). The same combination of these four drugs gives the estimation of OR=32.97 compared to docetaxel (the 10th findings in Table 2).

Top findings from Table 4 show the effect of adding either acetaminophen or zoledronate on existing drug combinations. For example, six findings are from adding zoledronate and the other four findings are from adding acetaminophen. This suggests further investigation on possible interactions between acetaminophen and zoledronate with other drugs.

### Interactive visualization of DDIs

The complexity of directional effects among high-order DDIs requires a concise yet comprehensive way to organize and present the complex relationships among interesting drug sets. We develop a visualization tool using sunburst diagram, inputting a drug set and visualizing directional DDIs among all its subsets in an interactive manner.

**Fig. 2:**
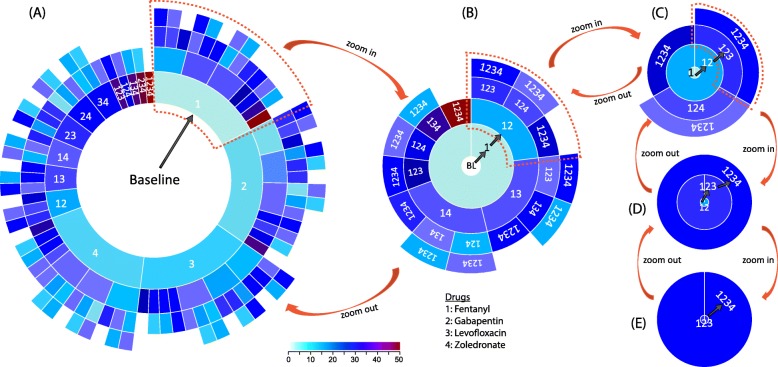
Interactive visualization for all possible directional DDI effects related to the subsets of fentanyl, gabapentin, levofloxacin and zoledronate. **a** shows the overall picture of all possible directional DDIs effects. **b** shows details of DDI effects of taking fentanyl as baseline, by zooming in the highlighted part of (**a**). **c** shows the details of DDI effects of taking both fentanyl and gabapentin as baseline, by zooming in the highlighted part of (**b**). **d** shows the details of DDI effects of taking fentanyl, gabapentin and levofloxacin as baseline, by zooming in the highlighted part of (**c**). **e** shows the DDI effects of taking all four drugs vs. taking the first three ones. The sector color indicates the effect size of DDIs from inner to outer ring sectors

Figure 2 shows an example of the visualization of a four-drug combination including fentanyl, gabapentin, levofloxacin and zoledronate. Due to the space limitation, we use numbers 1, 2, 3, and 4 to denote these four drugs. Figure 2a presents an overview the DDIs corresponding to four drugs, showing all paths from baseline node (i.e., none of drugs 1,…,4 are taken) to all the subsets of these four drugs. In the plot, circumjacent ring sectors present the directional DDI from inner sector to outer one. For example, the arrow in Fig. 2a represents the DDI from baseline to drug 1 (i.e., fentanyl), with the color representing the effect size (OR).

Given a user-interested drug, for example drug 1, we can zoom in to focus on only directional DDIs on top of drug 1. Figure 2b shows all the directional DDIs by adding subsets of other three drugs to drug 1. Iteratively, from Fig. 2b to c, we can zoom in to a two-drug combination (drug 1 and drug 2) to generate another plot for showing the local DDI results on top of drugs 1 and 2. Similarly, we can also zoom out from a plot of local DDIs to a global one, for obtaining a comprehensive overview of the interested sets of drugs (e.g., from Fig. 2e → d →c →b →a).

## Discussion

This analysis extends our previous work [7] from estimating DDI directional effects of up to three drugs with minimum support of 1000 to a larger scale involving higher-order combinations with less stringent minimum support of 250. In this paper, we investigate the risk of adding up to seven drugs at a time with minimum support of 250 on the same dataset. We employed an efficient Apriori implementation in R package “arules”, the same as that used in our previous work [7], to extract the frequent itemsets (MinSup=250) from the total 4,077,447 records. However, as we also need to extract the itemsets with MinSup=1 from myopathy records, the computational burden for more than three drug combinations would increase too dramatically to be affordable. In practice, we tried the previous implementation for extracting itemset with MinSup=1 on myopathy cases using Algorithm 1 on a Window 10 Enterprise 64 bit desktop with an Intel (R) Core(TM) i9-7900X CPU and 32 GB memory. We were unable to obtain drug combinations involving more than four drugs due to the huge number of drug combinations. In this work, using the newly proposed Algorithm 2, we were able to obtain all the drug combinations using MinSup=1, which involved up to seven drugs from myopathy cases. The newly designed tool also allows us to visualize the high-order directional DDI results effectively.

Tatoneti et al. [9] and Li et al. [10] also analyzed the FAERS data, but focused on examining either single drug effects or two-way drug interaction effects, without exploring the directional effects proposed here. As an exploratory study, our work focused on estimating the ORs of high-order directional DDIs on myopathy using the FAERS data. For validation purpose, we assessed the sensitivity of our findings using available side effect databases including OFFSIDES and TWOSIDES databases from [9] and Side Effect Resource (SIDER) database [11].

Our comparison with OFFSIDES focused on two events: myopathy toxic and myopathy steroids. In the OFFSIDES database, there are 17 drugs with myopathy toxic as event. Except potassium acetate, all the other 16 drugs exist in our data. Our method identified 14 out of 16 drugs with significant p-values, while ethambutol and epinephrine were not captured. In addition, there are 19 drugs with myopathy steroid as event in OFFSIDES. Except cetraxate and salina, all the other 17 drugs exist in our data. Our method identified 14 out of 17 drugs with significant p-values, and vinorelbine, voriconazole and bortezomib were not captured.

In the TWOSIDES database, we focused on the 2-drug combinations associated with muscle weakness, rhabdomyolysis, muscle disorder, muscle paresis, muscle spasm, muscle inflammation, musculoskeletal pain, myasthenia gravis, muscle strain, and muscle rupture. A total of 32,304 unique 2-drug combinations linking to events listed above were reported in the TWOSIDES database, among which 7,444 were identified in our “2-drug vs. baseline” results with significant p-values. The ORs of myopathy risk, based on our analysis, for these two-drug combinations ranging from 24.95 to 0.05. Specifically, the OR is 24.95 for (fulvestrant, gabapentin) with *p*= 3.89E-138, and the OR is 0.05 for (heparin, pancuronium) with *p*= 2.05E-07. Both are significant after Bonferroni correction.

For high-order findings, due to the lack of high-order DDI databases, we alternatively assessed the individual drugs from our high-order results with known myopathy-related drugs. For example, we compared the unique drugs reported in our “four-drugs vs. baseline” findings with the SIDER database [11]. In the SIDER database, 97 drugs are listed with myopathy as event, among which 75 drugs exist in our FAERS data. In our result, we have 27,191 four-drug combinations with significant p-values, which consist of 372 unique drugs. 37 out of 75 SIDER drugs are part of the identified 372 unique drugs. Of note, since we focus on identifying high order drug combinations that induce adverse effect, any individual drug from our reported drug combinations may not necessarily have an impact on the adverse effect by itself alone.

Our analysis has uncovered a number of interesting drug combinations leading to increased risk for myopathy. Drugs most often appearing in the top results are bisphosphonates (zolendronte, pamidronate), chemotherapy agents (doxorubicin, capecitabine, vincristine, cyclophosphamide), opioid analgesics (fentanyl, oxycodone), non-opioid analgesics (acetaminophen, gabapentin), corticosteroids (dexamethasone), and other renally-excreted drugs (levofloxacin, ciprofloxacin, and gadolidium based contrast agents). In the top 20 4- vs. 3-drug combinations, only zolendronate and acetaminophen added on top of 3-drug combinations led to increased risk of myopathy events. Interestingly, many of the co-prescribed medications are nephrotoxic or renally cleared, which may lead to pharmacokinetic-based drug interactions with other drugs. Additionally, many of drugs have reported myopathy or rhabdomyolysis risk. Thus, it is likely that interactions in pharmacodynamic mechanisms also increase risk of myopathy. For instance, each of the drugs in the top 4-way combination (fentanyl, gabapentin, levofloxacin, and zolendronate) have each individually been associated with myopathy [12–18].

Zolendronate and pamidronate are bisphosphonates used to treat osteoporosis, hypercalcemia of malignancy, Paget’s disease, and metastatic bone metastases. It is primarily cleared through renal excretion. Myalgia following infusion is reported in 65–70% of postmenopausal women but is typically self-limiting [12, 13]. While acetaminophen would most likely be associated with myopathy due to its use in treating pain, there are several reports of rhabdomyolysis as a symptom of acetaminophen overdose [19–21].

A limitation to the structured FAERS data is that it does not report timing of drug administration with respect to the adverse event. Thus, it is difficult to distinguish drugs used to treat myopathy from those that cause myopathy. Some drugs, such as opioid and non-opioid analgesics, muscle relaxers, and corticosteroids may have been used for treatment of myopathy. Thalidomide, cyclophosphamide and dexamethasone have been used in combination to treat sporadic late-onset nemaline myopathy with monoclonal gammopathy of undetermined significance (SLONM-MGUS) [22]. Thalidomide has also been reported as a treatment for scleromyxedema with myopathy [23]. However, glucocorticoids such as prednisone and dexamethasone are well-known as a cause of drug-induced myopathy, especially at high doses [24]. Opioids (e.g. fentanyl, oxycodone) are also associated with myopathy events and non-opioid analgesics such as celecoxib and ibuprofen are nephrotoxic, which could increase risk of myopathy due to other myotoxic drugs. Thus, it is difficult to distinguish whether these agents instigated the myopathy adverse events or were used in its treatment.

## Conclusions

We have proposed a high-order directional DDI mining strategy for identifying myopathy associated drug interactions from large-scale ADE reporting database. We have demonstrated its efficiency using real data from the public health record database FAERS. Our method confirms several prior drug or DDI effects on myopathy, as well as suggests novel interactions involving more than three drugs. We have also developed a more effective and scalable visualization tool for easy interpretation of DDI findings. However, the absence of report timing of drug administration with respect to the adverse event limits our capability to distinguish DDI findings from causal to treatment. Given this limitation, this work can be further expanded towards including temporal relation information between drug administration and event, to improve the inference of causal DDI effects.

## Supplementary information

**Additional file 1** Supplementary materials. Table S1-S7 — Top 10 OR results for taking 1-7 drug versus baseline. Top 10 OR results for taking one to seven drugs vs. baseline: The OR is based on the frequent drug combinations as resulted using the Apriori algorithm with *MinSup*=250.

## Data Availability

The data used for this analysis included reports from FAERS collected between Q1 2004 and Q3 2012. FAERS is a publicly available database that contains information on adverse event and medication error reports submitted to FDA.

## Abbreviations

ADE: Adverse drug event
D3: Data-driven documents
DC: Drug combination
DDI: Drug-drug interaction
FAERS: FDA adverse event reporting system
FDA: Food and drug administration
OR: Odds ratio
Q1: First quarter
Q3: Third quarter
SIDER: Side effect resource
