# Supplementary material for: Mining and visualizing high-order directional drug interaction effects using the FAERS database
Source: BMC Med Inform Decis Mak. 2020 Mar 18;20(Suppl 2):50. doi: 10.1186/s12911-020-1053-z (PMC7079342; doi:10.1186/s12911-020-1053-z)
Supplement: Supplementary file 1 — Additional file 1 Supplementary materials. Table S1-S7 — Top 10 OR results for taking 1-7 drug versus baseline. Top 10 OR results for taking one to seven drugs vs. baseline: The OR is based on the frequent drug combinations as resulted using the Apriori algorithm with MinSup=250. [file 12911_2020_1053_MOESM1_ESM.pdf]

Supplementary Materials for "Mining and visualizing high-order directional drug interaction effects using the FAERS database" by Xiaohui Yao, Tiffany Tsang, Qing Sun, Sara K. Quinney, Pengyue Zhang, Xia Ning, Lang Li, Li Shen.

Supplementary Results: Top DDIs

Below we present the top 10 findings from one to seven drugs versus the baseline. The use of $MinSup = 250$ allows us to identify more myopathy risk DDIs than our prior study [1], consequently to help provide more comprehensive references for adverse effects of DDIs. New findings from our analysis are highlighted in bold in Table S1-S7.

Table S1 Top 10 OR results for taking one drug vs. baseline: The OR is based on the frequent drug combinations as resulted using the Apriori algorithm with $MinSup = 250$. The p-value significance is based on the Bonferroni corrected threshold 4.86E-05.

Rank	Drug	Odds Ratio	p-Value
1	**Fusidic Acid**	27.24	1.02E-308
2	**Telbivudine**	12.94	3.60E-128
3	**Cerivastatin**	12.40	7.38E-66
4	**Trabectedin**	6.26	6.41E-22
5	**Terconazole**	6.25	2.69E-22
6	Gadoteridol	5.57	2.88E-128
7	Gadoversetamide	5.49	1.05E-149
8	Chlorhexidine	5.48	1.20E-214
9	**Flucloxacillin**	5.44	7.26E-52
10	**Pindolol**	5.37	1.48E-32

Table S2 Top 10 OR results for 2-drug combination vs. baseline: The OR is based on the frequent drug combinations as resulted using the Apriori algorithm with $MinSup = 250$. The p-value significance is based on the Bonferroni corrected threshold 1.86E-06.

Rank	2-drug combination	OR	p-Value
1	**Fulvestrant, Levofloxacin**	33.22	5.09E-142
2	**Gabapentin, Gadobenate Dimeglumine**	28.16	1.35E-115
3	**Doxycycline, Pamidronate**	25.32	3.48E-131
4	**Esomeprazole, Fulvestrant**	25.31	2.13E-118
5	**Fulvestrant, Gabapentin**	24.95	3.89E-138
6	**Nystatin, Pamidronate**	24.14	4.32E-144
7	**Alprazolam, Fulvestrant**	24.01	6.89E-98
8	**Pamidronate, Rabeprazole**	23.99	1.02E-113
9	**Gadopentetate Dimeglumine, Mycophenolate Mofetil**	23.05	1.33E-100
10	**Nitroglycerin, Pamidronate**	22.98	2.00E-105

Table S3 **Top 10 OR results for 3-drug combination vs. baseline: The OR is based on the frequent drug combinations as resulted using the Apriori algorithm with $MinSup = 250$. The p-value significance is based on the Bonferroni corrected threshold 7.88E-7.**

Rank	3-drug combination	OR	p-Value
1	**Gabapentin, Iron, Zoledronate**	39.0	2.99E-158
2	**Atorvastatin, Prochlorperazine, Zoledronate**	37.64	7.47E-154
3	**Gabapentin, Hydromorphone, Pamidronate**	36.61	4.28E-186
4	**Acetaminophen, Fulvestrant, Gabapentin**	36.28	7.22E-141
5	**Metoprolol, Pamidronate, Zolpidem**	35.78	1.99E-138
6	**Atorvastatin, Ciprofloxacin, Oxycodone**	34.83	2.56E-138
7	**Azithromycin, Gabapentin, Pamidronate**	33.03	5.59E-177
8	**Acetaminophen, Letrozole, Levofloxacin**	32.19	1.54E-142
9	**Docetaxel, Gabapentin, Oxycodone**	32.12	5.13E-136
10	**Chlorhexidine, Diazepam, Zoledronate**	31.94	7.38E-124

Table S4 **Top 10 OR results for 4-drug combination vs. baseline: The OR is based on the frequent drug combinations as resulted using the Apriori algorithm with $MinSup = 250$. The p-value significance is based on the Bonferroni corrected threshold 1.50E-6.**

Rank	4-drug combination	OR	p-Value
1	**Fentanyl, Gabapentin, Levofloxacin, Zoledronate**	49.65	6.46E-194
2	**Furosemide, Gabapentin, Levofloxacin, Zoledronate**	48.43	2.66E-174
3	**Azithromycin, Ciprofloxacin, Levofloxacin, Zoledronate**	45.3	2.27E-157
4	**Azithromycin, Gabapentin, Levofloxacin, Zoledronate**	44.97	4.17E-159
5	**Gabapentin, Levofloxacin, Omeprazole, Zoledronate**	44.76	4.47E-180
6	**Gabapentin, Levofloxacin, Zoledronate, Zolpidem**	44.74	2.77E-214
7	**Fentanyl, Levofloxacin, Omeprazole, Zoledronate**	44.62	6.06E-181
8	**Gabapentin, Levofloxacin, Pamidronate, Zolpidem**	44.37	1.10E-179
9	**Alprazolam, Levofloxacin, Omeprazole, Oxycodone**	43.24	1.45E-151
10	**Gabapentin, Levofloxacin, Omeprazole, Oxycodone**	42.44	8.54E-160

Table S5 **Top 10 OR results for 5-drug combination vs. baseline: The OR is based on the frequent drug combinations as resulted using the Apriori algorithm with $MinSup = 250$. The p-value significance is based on the Bonferroni corrected threshold 7.54E-6.**

Rank	5-drug combination	OR	p-Value
1	**Acetaminophen, Fentanyl, Levofloxacin, Omeprazole, Zoledronate**	54.34	9.84E-175
2	**Gabapentin, Levofloxacin, Oxycodone, Zoledronate, Zolpidem**	51.7	7.77E-174
3	**Gabapentin, Levofloxacin, Pamidronate, Zoledronate, Zolpidem**	50.03	6.06E-185
4	**Gabapentin, Hydrocodone, Levofloxacin, Oxycodone, Zoledronate**	46.41	2.02E-165
5	**Hydrocodone, Levofloxacin, Omeprazole, Oxycodone, Zoledronate**	44.84	4.34E-171
6	**Acetaminophen, Levofloxacin, Omeprazole, Zoledronate, Zolpidem**	44.5	1.04E-161
7	**Gabapentin, Omeprazole, Oxycodone, Zoledronate, Zolpidem**	43.38	1.10E-156
8	**Acetaminophen, Gabapentin, Levofloxacin, Oxycodone, Zoledronate**	43.35	1.40E-231
9	**Fentanyl, Gabapentin, Morphine, Oxycodone, Zoledronate**	41.12	2.79E-173
10	**Furosemide, Levofloxacin, Oxycodone, Potassium Chloride, Zoledronate**	40.74	2.52E-146

Table S6 Top 10 OR results for 6-drug combination vs. baseline: The OR is based on the frequent drug combinations as resulted using the Apriori algorithm with $MinSup = 250$. The p-value significance is based on the Bonferroni corrected threshold 1.06E-4.

Rank	6-drug combination	OR	p-Value
1	**Acetaminophen, Gabapentin, Hydrocodone, Levofloxacin, Oxycodone, Zoledronate**	46.41	2.02E-165
2	**Acetaminophen, Hydrocodone, Levofloxacin, Omeprazole, Oxycodone, Zoledronate**	45.26	1.76E-171
3	**Acetaminophen, Dexamethasone, Gabapentin, Omeprazole, Oxycodone, Zoledronate**	39.5	4.10E-146
4	**Acetaminophen, Gabapentin, Levofloxacin, Oxycodone, Pamidronate, Zoledronate**	38.9	5.30E-163
5	**Acetaminophen, Fentanyl, Hydrocodone, Levofloxacin, Oxycodone, Zoledronate**	38.59	1.61E-166
6	**Acetaminophen, Dexamethasone, Levofloxacin, Omeprazole, Oxycodone, Zoledronate**	38.43	4.78E-145
7	**Acetaminophen, Furosemide, Hydrocodone, Levofloxacin, Oxycodone, Zoledronate**	37.02	1.54E-144
8	**Acetaminophen, Levofloxacin, Omeprazole, Oxycodone, Pamidronate, Zoledronate**	36.2	2.93E-151
9	**Acetaminophen, Furosemide, Levofloxacin, Oxycodone, Pamidronate, Zoledronate**	34.21	1.50E-138
10	**Acetaminophen, Furosemide, Gabapentin, Hydrocodone, Oxycodone, Zoledronate**	34.18	3.09E-157

Table S7 Top 10 OR results for 7-drug combination vs. baseline: The OR is based on the frequent drug combinations as resulted using the Apriori algorithm with $MinSup = 250$. The p-value significance is based on the Bonferroni corrected threshold 4.55E-3.

Rank	7-drug combination	OR	p-Value
1	**Acetaminophen, Dexamethasone, Hydrocodone, Omeprazole, Oxycodone, Zoledronate, Zolpidem**	28.41	4.78E-115
2	**Acetaminophen, Dexamethasone, Hydrocodone, Levofloxacin, Oxycodone, Pamidronate, Zoledronate**	27.98	2.24E-115
3	**Acetaminophen, Fentanyl, Hydrocodone, Morphine, Oxycodone, Zoledronate, Zolpidem**	24.81	8.77E-106
4	**Acetaminophen, Dexamethasone, Hydrocodone, Oxycodone, Pamidronate, Zoledronate, Zolpidem**	21.34	3.27E-88
5	**Epoetin Alfa, Gadobenate Dimeglumine, Gadodiamide, Gadopentetate Dimeglumine, Gadoteridol, Gadoversetamide, Metoprolol**	17.71	8.17E-74
6	**Epoetin Alfa, Gadobenate Dimeglumine, Gadodiamide, Gadopentetate Dimeglumine, Gadoteridol, Gadoversetamide, Warfarin**	17.54	6.51E-81
7	**Acetaminophen, Amoxicillin, Clavulanate, Oxycodone, Pamidronate, Potassium, Zoledronate**	16.69	2.38E-76
8	**Epoetin Alfa, Gadobenate Dimeglumine, Gadodiamide, Gadopentetate Dimeglumine, Gadoteridol, Gadoversetamide, Sevelamer**	16.4	4.07E-85
9	**Acetaminophen, Fentanyl, Hydrocodone, Morphine, Oxycodone, Pamidronate, Zoledronate**	15.57	1.51E-63
10	**Acetaminophen, Amoxicillin, Clavulanate, Hydrocodone, Oxycodone, Potassium, Zoledronate**	8.96	4.69E-33

Author details

References

1. Chasioti, D., Yao, X., Zhang, P., Lerner, S., Quinney, S.K., Ning, X., Li, L., Shen, L.: Mining directional drug interaction effects on myopathy using the FAERS database. IEEE Journal of Biomedical and Health Informatics, 1–1 (2018)
